# DGCA: A comprehensive R package for Differential Gene Correlation Analysis

**DOI:** 10.1186/s12918-016-0349-1

**Published:** 2016-11-15

**Authors:** Andrew T. McKenzie, Igor Katsyv, Won-Min Song, Minghui Wang, Bin Zhang

**Affiliations:** 1 Department of Genetics and Genomic Sciences, Icahn School of Medicine at Mount Sinai, One Gustave L. Levy Place, New York, NY 10029 USA; 2 Icahn Institute of Genomics and Multiscale Biology, Icahn School of Medicine at Mount Sinai, One Gustave L. Levy Place, New York, NY 10029 USA; 3 Medical Scientist Training Program, Icahn School of Medicine at Mount Sinai, One Gustave L. Levy Place, New York, NY 10029 USA; 4 Department of Genetics & Genomic Sciences, Icahn School of Medicine at Mount Sinai, 1470 Madison Avenue, Room S8-111, New York, NY 10029 USA

**Keywords:** Differential correlation, Differential coexpression, Multiscale clustering analysis, R package, RNA-Seq, TP53, Breast cancer, Triple negative breast cancer

## Abstract

**Background:**

Dissecting the regulatory relationships between genes is a critical step towards building accurate predictive models of biological systems. A powerful approach towards this end is to systematically study the differences in correlation between gene pairs in more than one distinct condition.

**Results:**

In this study we develop an R package, DGCA (for Differential Gene Correlation Analysis), which offers a suite of tools for computing and analyzing differential correlations between gene pairs across multiple conditions. To minimize parametric assumptions, DGCA computes empirical *p*-values via permutation testing. To understand differential correlations at a systems level, DGCA performs higher-order analyses such as measuring the average difference in correlation and multiscale clustering analysis of differential correlation networks. Through a simulation study, we show that the straightforward z-score based method that DGCA employs significantly outperforms the existing alternative methods for calculating differential correlation. Application of DGCA to the TCGA RNA-seq data in breast cancer not only identifies key changes in the regulatory relationships between *TP53* and *PTEN* and their target genes in the presence of inactivating mutations, but also reveals an immune-related differential correlation module that is specific to triple negative breast cancer (TNBC).

**Conclusions:**

DGCA is an R package for systematically assessing the difference in gene-gene regulatory relationships under different conditions. This user-friendly, effective, and comprehensive software tool will greatly facilitate the application of differential correlation analysis in many biological studies and thus will help identification of novel signaling pathways, biomarkers, and targets in complex biological systems and diseases.

**Electronic supplementary material:**

The online version of this article (doi:10.1186/s12918-016-0349-1) contains supplementary material, which is available to authorized users.

## Background

**Fig. 1:**
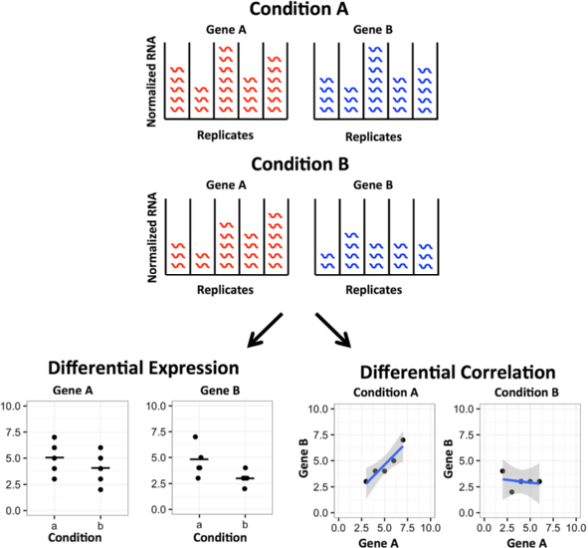
An example demonstrating the theoretical difference between differential expression and differential correlation. The top panel shows the RNA expression levels for two example genes in two example conditions. The bottom left panel shows that both of these genes have decreased expression values from condition A to condition B. On the other hand, the bottom right panel shows that these genes have positive correlation in condition A but no correlation in condition B, which could not have been predicted on the basis of the differential expression relationships alone

Over the past two decades, a wealth of high-dimensional biological data types have emerged including microarray, RNA-seq, proteomics, epigenomics, metabolomics, lipidomics, and many others [1, 2]. A common use of these data is to gather and compare samples from multiple conditions, e.g., disease and non-diseased, in an attempt to identify molecular identifiers (e.g., probes, transcripts, genomic features, proteins, metabolites, lipids; henceforth, “genes”) that distinguish between different conditions. Currently, the most common method of comparing samples from different conditions is differential expression analysis [3, 4]. Recently, new methods for detecting differential co-expression or differential correlation analysis have emerged to gain insights into the difference in gene-gene relationships between various conditions of interest. Distinct from differential expression, differential correlation operates on the level of gene pairs rather than individual genes (Fig. 1). Differential co-expression analysis can start with coexpressed gene modules or clusters based on the similarity of their gene expression in each condition using WGCNA [5] and MEGENA [6] and then computes module overlap statistics between conditions [7] or the average modular differential connectivity [8, 9]. Alternative approaches including DICER [10], DINGO [11], CoXpress [12], SDC [13], DiffCoEx [14], GSCA [15], and GSNCA [16] were developed to identify differential co-expression relationships between conditions and gene modules in each condition simultaneously.

While differential coexpression analysis has proven useful in identifying significantly different modular connectivity patterns, differential correlation analysis of individual gene pairs is far more granular. As an example of such a differential correlation, RNA levels of the prostate cancer biomarker gene *AMACR* have been found to have positive correlation with the tumor suppressor gene *PTEN* in adjacent normal tissue samples, but not in prostate cancer tissue samples [17]. Multiple approaches for identifying differential correlation between individual gene pairs have been developed, including DiffCorr [18], EBcoexpress [19], and Discordant [20]. DiffCorr calculates correlations in each condition and uses the difference in z-transformed correlation coefficients to calculate *p*-values. EBcoexpress uses an empirical Bayesian approach and a nested expectation-maximization algorithm to estimate the posterior probability of differential correlation between gene pairs. Discordant fits a mixture distribution of correlation classes in each condition and uses an expectation-maximization algorithm to estimate the posterior probability of each differential correlation category [21].

In this manuscript, we introduce DGCA, an R package to identify differential correlations between gene pairs in multiple conditions. DGCA shares some features with existing approaches for identifying differential correlation. Like DiffCorr, DGCA transforms correlation coefficients to z-scores and uses differences in z-scores to calculate *p*-values of differential correlation between genes. Like Discordant, DGCA classifies differentially correlated gene pairs into the nine possible categories. However, DGCA differs from the existing differential correlation approaches in four key ways. First, DGCA calculates false discovery rate of differential correlations through non-parametric sample permutation. Second, DGCA can calculate the average difference in correlation between one gene and a gene set across two conditions. Third, DCGA integrates with MEGENA to perform multiscale clustering analysis of differential correlation networks to identify gene modules (clusters) and hub genes. Finally, DGCA provides comprehensive downstream functional analysis of differential correlation structures including visualization, gene ontology (GO) enrichment, and network tools.

To assess the performance of DGCA and the existing methods EBcoexpress and Discordant in identifying differentially correlated gene pairs, we designed and implemented a simulation study. Next, we applied DGCA to the breast cancer data from The Cancer Genome Atlas (TCGA) with and without p53 and PTEN coding mutations. We identified five genes with a significant change of correlation with *TP53* in the p53-mutated samples, and two genes with a significant change of correlation with *PTEN* in the PTEN-mutated samples. We showed that each gene’s differential correlation with *TP53/PTEN* between p53/PTEN wildtype and inactivated samples is uncorrelated with its differential expression in this data set. By evaluating differential correlations between the overall correlation matrices, DGCA allowed us to harness additional insights about the regulatory patterns among *TP53*’s targets following p53 mutation. We further performed DGCA on the estrogen receptor-positive (ER+) and triple negative (TN) breast cancer subtypes in the TCGA breast cancer data and identified key gene ontology categories that differ in regulation between breast cancer subtypes. By integrating DGCA with the multiscale clustering approach MEGENA, we identified modules containing key hub genes that coordinate differential correlations between the two subtypes. We demonstrated that DGCA/MEGENA can better detect modules than the established approaches DICER and DiffCoEx in another simulation study. Furthermore, we showed that a majority of the modules detected by DGCA/MEGENA in the TCGA breast cancer data were not detected by DiffCoEx or DICER, while a majority of the modules detected by either DiffCoEx or DICER were uncovered by DGCA/MEGENA, revealing the novelty of our proposed module detection approach.

## Methods

### Differential correlation analysis flow

**Fig. 2:**
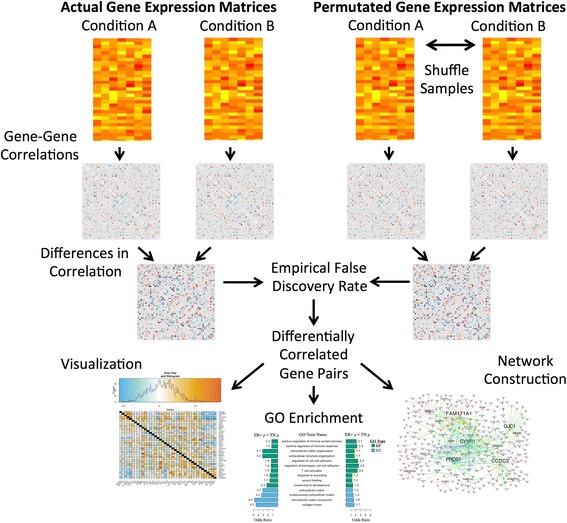
Workflow for the Differential Gene Correlation Analysis (DGCA) R package. Users input a gene expression matrix, a design matrix to specify the conditions, and a comparison vector to specify which conditions will be compared. DGCA then calculates the gene pair correlations within each condition, processes these correlation values, and compares them to build up a difference in correlation matrix. If permutation testing is chosen, DGCA will perform the same procedure on permuted gene expression matrices. These permutation samples are used to estimate an empirical false discovery rate. After investigators choose the significance threshold for differential correlation between conditions (if any) to choose downstream gene pairs, they can use DGCA’s capacities for visualization, gene ontology (GO) enrichment, and/or network construction

DGCA has three main inputs including a matrix of gene expression values, a design matrix specifying conditions associated with samples, and a specification of the conditions for comparison (Fig. 2). Prior to the actual analysis, users have the option to filter the input expression matrix to remove genes with low expression central tendency and/or dispersion, since these genes are more likely to have spurious correlations. Note that central tendency refers to measures of centrality in a distribution, including the arithmetic mean or median, while dispersion refers to measures of spread in a distribution, including the standard deviation and the dispersion index (the variance divided by the mean). To stabilize the variance of sample correlation coefficients in each condition, the Fisher z-transformation is employed [22, 23]:

$$ z= atanh(r)=\frac{1}{2}lo{\mathit{\mathsf{g}}}_e\left(\frac{1+r}{1-r}\right) $$

where *r* is the sample correlation coefficient, *log*_*e*_ is the natural logarithm function, and *atanh* is the arc-tangent hyperbolic function. In this context, the Fisher z-transformation function serves as a normalizing transformation. The variance of the resulting z-scores depends on whether the sample correlation coefficient is the Pearson product-moment correlation coefficient (r_p_) or the Spearman’s rank correlation coefficient (r_s_) [24]. When the underlying distribution is assumed to be bivariate normally distributed, the variance can be calculated by

$$ var\left({r}_p\right)=\frac{1}{n-3}\mathrm{or}\;var\left({r}_s\right)=\frac{1.06}{n-3} $$

where *n* is the sample size of the calculated correlation. Notably, the variance of the correlation coefficients in a particular condition could differ due to a different number of samples or due to missing data in one or both of the genes’ expression measurements. These equations for the variance have been found to be valid over a wide range of sample sizes that are common in current biological data sets [24]. Due to the denominator, the equations require that there are at least 4 samples in each condition considered. The difference in z-scores (*dz*) between two conditions can then be calculated by,

$$ dz=\frac{\left({z}_1-{z}_2\right)}{\sqrt{\left|{s}_{z_1}^2-{s}_{z_2}^2\right|}} $$

where ${s}_{z_x}^2$ refers to the variance of the z-score in condition x. Using the difference in z-scores *dz*, a two-sided *p*-value can be calculated using the standard normal distribution. Gene pairs can then be ranked on the basis of their relative strength of differential correlation.

### Multiple hypothesis testing correction

When testing for differential correlation between gene pairs in genome-wide experiments, the number of hypothesis tests grows quadratically in the number of genes. For example, differential correlation analysis of 20,000 genes would require 199,990,000 hypothesis tests. Therefore, DGCA offers several options for adjusting *p*-values for multiple hypothesis tests, including the conservative Benjamini-Hochberg *p*-value adjustment method [25, 26] and the local false discovery rate method [27]. However, even when using these options, it can be difficult to make intuitive sense of the *p*-values returned because the *p*-values are originally derived from the difference of z-scores method, which depends on specifying the correct form for the variance of the sample correlation coefficients, and in turn on the bivariate distribution of the gene expression values. Therefore, DGCA also offers to generate permutation samples by randomly shuffling the sample labels across the input conditions and then re-computing the differential correlation calls. The z-scores from the original and permuted data sets are used to calculate empirical *p*-values, using a reference pool distribution approach adapted from the R package qvalue [28]. These empirical *p*-values are used to estimate the proportion of null hypotheses in empirical *p*-values by extrapolating a linear trend from a cubic spline fitted over candidate ranges of the tuning parameter lambda [29]. Then, q-values are calculated based on the empirical *p*-values and the estimated proportion of null hypotheses.

### Classifying differentially correlated gene pairs

**Fig. 3:**
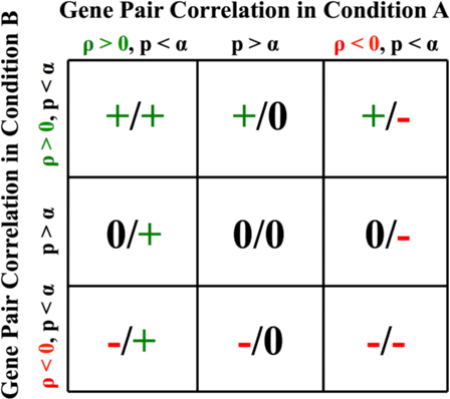
Definition of differential correlation classes. This diagram demonstrates the definition of differential correlation classes used throughout DGCA in the case of two conditions. The default *p*-value significance level threshold (α) parameter setting for the hypothesis test of non-zero correlation in one individual condition in DGCA and throughout this manuscript is 0.05, although users of DGCA can adjust this parameter if they choose to. In each condition, gene pairs are defined as having a non-significant correlation (*p*-value < α), a significant and positive correlation (*p*-value < α, correlation (ρ) > 0), or a significant and negative correlation (*p*-value < α, correlation (ρ) < 0). The Cartesian product of the 3 possible correlation classes in one condition with the 3 in the other condition yields a total of 9 possible differential correlation classes. Note that, theoretically, a gene pair with a significant positive correlation in one condition and a non-significant correlation in another condition may not be significantly differentially correlated between these conditions since the correlation class identification is independent of the differential correlation hypothesis test

At the most basic level, gene pairs can be classified as having gain of correlation (GOC) or loss of correlation (LOC) between one condition compared to another. For example, a gene pair with ρ = 0.8 in a condition A and ρ = 0.2 in a condition B is defined as having a gain of correlation in the condition A and a loss of correlation in the condition B. To go beyond this binary classification, we also determine if two genes are significantly correlated in each condition or not. By default and throughout this manuscript, the α threshold for statistical significance of the hypothesis test that the correlation in each condition is significantly different from zero is defined as *p* < 0.05, although users can set different thresholds. This *p*-value is calculated based on the approximation that the correlation coefficient follows a t-distribution with *n* - 2 degrees of freedom, where *n* is the sample size of the calculated correlation [30]. The *p*-values associated with the hypothesis test of non-zero correlation in each separate condition are not adjusted for multiple tests by default. Based upon a threshold for correlation significance and the sign of correlation in each condition (i.e., positive or negative), gene-gene correlations in each condition can be categorized into 3 classes, i.e. significant positive correlation, no significant correlation, and significant negative correlation. Therefore, there are 9 classes for differential correlations between two conditions (Fig. 3). DGCA also allows users to perform downstream analyses of differential correlation classes, including heatmap visualization and gene ontology (GO) enrichment analysis of the genes in each differential correlation class.

### Calculating the average differential correlation between gene pairs

It is sometimes useful to measure the difference in average correlations of a gene and a set of genes between two conditions. DGCA quantifies the median difference in z-transformed correlation coefficients of a gene and a gene set (henceforth, median difference in z-score) between two conditions. In this context, a median difference in z-score above 0 indicates a tendency towards a gain of correlation between the given gene and the gene set in the first condition with respect to the second condition, while a median difference in z-score below 0 indicates a tendency towards a loss of correlation. To measure the significance of the median change in correlation, DGCA leverages the permutation samples to calculate empirical *p*-values, as illustrated by the following equation,

$$ 1-{\displaystyle \sum_p^n\left|{}_{\mathrm{j}:\mathrm{j}\ne \mathrm{i}}^{med}\left(d{z}_{ij}\right)\right|}>\left|{}_{\mathrm{j}:\mathrm{j}\ne \mathrm{i}}^{med}\left(d{z}_{ij}^p\right)\right|/n $$

where, *med* refers to the function for the median of a set, *i* is the gene for which the median difference in z-score is being compared between conditions, *dz*_*ij*_ is the difference in z-transformed correlation coefficients between genes *i* and *j* in the two conditions, *p* refers to a permutation, and *n* refers to the total number of permutations. As a generalization, DGCA also offers to calculate the median difference in z-score between all gene pairs in two conditions. In this case, in order to calculate a two-sided *p*-value, the median is taken over all of the gene pairs using the following equation,

$$ 1-{\displaystyle \sum_p^n\left|{}_{\mathrm{i},\mathrm{j}:\mathrm{i}\ne \mathrm{j}}^{med}\left(d{z}_{ij}\right)\right|>\left|{}_{\mathrm{i},\mathrm{j}:\mathrm{i}\ne \mathrm{j}}^{med}\left(d{z}_{ij}^p\right)\right|}/n $$

This approach is similar to the modular differential correlation calculation that was previously described and used [8, 9].

### Simulated biological data and comparison with EBcoexpress and Discordant

We designed a simulation study to assess the performance of DGCA and the existing methods EBcoexpress (version 1.12.0) [19] and Discordant (version 0.99.0) [20] at detecting differentially correlated gene pairs in the presence of noise. We did not compare DGCA to DiffCorr [18] in the simulation study, since both of these packages use the Fisher z-transformation and z-score calculation as its underlying algorithm, although DGCA offers a number of additional options, including permutation testing to quantify the statistical significance of gene-gene differential correlation. Among the 600 genes in our simulation study, 300 have high average expression and high dispersion (i.e., are occasionally “activated” in a biological sense) and 100 have high average expression and one-fold lower dispersion (i.e., are “housekeeping” genes that are constitutively expressed), and 200 have substantially lower average expression and high dispersion (i.e., genes that in a particular cell or tissue type are “non-expressed”). We built two covariance matrices to describe the dependence structure of the 179,700 gene pairs in each condition. We specified these covariance matrices so that 19 gene pairs, for which both of the genes were in the “activated” gene set, were segregated into each of the 8 differential correlation classes. Note that by default all of the gene pairs in the covariance matrices are set as 0 and the “0/0” differential correlation class means that two correlations under two conditions are not statistically different and thus it are not specified. To maintain a positive-definite covariance matrix in the case of negative correlations, we only set the super- and subdiagonals of the off-diagonals of the covariance matrix to non-zero values. The super- and subdiagonals were set to 0.5 or -0.5 times the variance of a particular gene pair, corresponding to positive or negative correlation in one condition, respectively. Because two differential correlation classes, ρ = +/+ and ρ = -/-, do not specify a difference between correlation between conditions, the number of actually differentially correlated gene pairs is reduced from 152 to 114. We then simulated the mean values for each gene using a negative binomial distribution with a dispersion parameter of 0.5, and set the mean values of less than one to one. The negative binomial distribution and its parameters were designed to approximately match the bimodality of expression means observed in RNA expression experiments [31, 32]. With the mean values for each gene as well as the covariance matrix, we simulated multivariate normally distributed gene expression matrices for various numbers of samples using the R package MASS [33]. We then employed receiver operating characteristic (ROC) curves [34] to measure the performance of DGCA and EBcoexpress on detecting the 114 truly differentially correlated gene pairs out of the total 179,700 gene pairs, for six different numbers of simulated samples in each condition, i.e., *n* = 10, 30, 50, 70, 90, and 100. We ran the simulation 5 times in order to estimate the standard error of the area under curve (AUC) statistics in the ROC curves. To estimate the statistical significance of the difference in performance between these methods, we used Student’s *t*-test to compare their AUCs at various sample sizes. We adjusted the *p*-value threshold required to call a comparison as significantly different for the number of simulated cases (*n* = 6; *p* < 0.05/6 = 0.00833).

Of the six types of differential correlation in this simulation study, 38 truly differentially correlated gene pairs had a strong difference (|Δρ| = 1) in correlation coefficient between conditions, while 76 had a medium difference (|Δρ| = 0.5) in correlation coefficient between conditions. We measured the accuracy of each of the methods to distinguish truly differentially correlated gene pairs for both the strong and medium difference classes, using the same comparison approach as in the full simulation experiment. For the simulations with *n* = 30 simulated samples, we also plotted representative ROC curves for each class, to visualize the ability of each of the differential correlation R packages to detect truly differentially correlated gene pairs in each of the 6 differential correlation classes. R code for this simulation study is available online (see “Availability and requirements”).

### Data processing for applications using breast cancer RNA expression data

We applied DGCA to the breast cancer RNA-seq data from The Cancer Genome Atlas (TCGA) [35] under two scenarios: a) the samples with and without a p53/PTEN mutation and b) the estrogen receptor-positive (ER+) samples and the triple negative (TN) samples. The level 3 RNAseqV2 data of breast cancer in the TCGA data portal first went through a log(x + 1) transform, then was quantile-normalized, and finally was corrected for age, batch, race, and gender using a linear regression approach. For the differential correlation analysis of p53/PTEN, this RNA expression data was filtered to contain only genes previously described as within p53/PTEN pathways (described below), but underwent no expression filtering. For the differential correlation analysis between ER+ and triple negative breast cancer, RNA expression data was filtered to remove genes in the bottom 25^th^ percentile of median expression and/or dispersion index of expression (described below), but underwent no filtering for specific gene sets.

### Differential correlation of p53/PTEN targets in normal and p53-/PTEN-mutated breast cancer tumor samples

Somatic mutation data was obtained from TCGA-curated mutations (Washington University School of Medicine curated mutation calling). We identified a set of RNA expression samples with and without a) a non-silent p53 DNA mutation and b) a non-silent PTEN DNA mutation. For the p53 analysis, we next filtered these RNA expression matrices to contain only genes that contain a p53 response element [36], are in the Molecular Signatures Database (MSigDB) p53 hallmark gene set [37], and/or are a downstream target of p53 in the Pathway Interaction Database [38]. For the PTEN analysis, we filtered the RNA expression matrices to only contain genes that are in the Biocarta PTEN pathway [39], the Sigma-Aldrich PTEN pathway [40], and/or have been identified in physical interactions with PTEN by affinity capture, as curated via Biogrid [41]. We then calculated the differential Spearman correlations on this filtered RNA expression matrices between the p53/PTEN mutated and non-p53/non-PTEN mutated breast cancer samples. For p53 only, we used the signType argument in DGCA to restrict the difference in z-score calculation between conditions to positive correlations, since p53 is a transcriptional activator and we reasoned that negative correlations with *TP53* are less likely to be biologically meaningful. Notably, the signType argument in DGCA, which refers to the differential correlation sign type (i.e., direction), can also be used to restrict the difference in z-score calculation between conditions to negative correlations if users are interested in only considering negative correlations as non-zero when assessing the difference in correlation between conditions. To assess the significance of the difference in gene-gene correlations, we generated empirical *p*-values via 10,000 permutations. For p53, we further visualized the global difference in correlations among p53-associated genes using the DGCA heatmap visualization, which builds on the gplots R package (version 2.17). We then visualized the gene-gene correlation matrix in each condition, and calculated both the median change in correlation for each gene and the median change in correlation for all gene pairs between the conditions, using 1000 permutations to quantify the significance.

### Differential correlations in RNA expression data from estrogen receptor-positive compared to triple-negative breast cancer samples

For the data from the ER+ and TN breast cancer samples, we removed the genes in the bottom 25^th^ percentile of median expression and/or dispersion index of expression. Note that the dispersion index is calculated as the ratio of the variance divided by the mean. We measured the differences in Pearson correlation between all pairs, using 5 permutations to quantify the significance of the difference in correlation, and identified genes with q-value < 0.05 for downstream analysis. Upon identifying the significantly differentially correlation gene pairs, we first collapsed these gene pairs to identify the genes uniquely present in gene pairs with a gain of correlation in one condition compared to the other, and used the DGCA wrapper function to perform gene ontology (GO) enrichment analysis based on the GOstats R package (version 2.34) [42] and org.Hs.eg.db GO annotation R package (version 3.1.2). For the GO enrichment analysis, we used genes identified in gene pairs with q < 0.01, since this greater specificity yielded a larger set of genes uniquely present in only one of the two differential correlation conditions. To compare the GO enrichment between the two conditions, we first filtered for those GO terms with between 50 and 600 gene symbols with a nominal significant *p*-value (<0.05) in at least one of the conditions. We next took the log of the ORs, since log ORs converge more rapidly to a normal distribution. We then calculated the standard error for the log ORs in each condition, using the following equations [43],

$$ \mathrm{OR}= \log \left(\frac{n_{11}\ast {n}_{22}}{n_{12}\ast {n}_{21}}\right) $$

$$ S{E}_{OR}=\sqrt{\frac{1}{n_{11}}+\frac{1}{n_{12}}+\frac{1}{n_{21}}+\frac{1}{n_{22}}} $$

where, n_11_ is the number of genes in the intersection between a gene signature and a GO term, n_12_ is the number of member genes in the GO term but not in the gene signature, n_21_ is the number of genes in the signature but not in the GO term, and n_22_ is the number of genes in the universe but not in either group. In order to quantify the significance of the difference in the log ORs in each group, we used the following equation,

$$ z=\frac{O{R}_1-O{R}_2}{\sqrt{S{E}_{O{R}_1}-S{E}_{O{R}_2}}} $$

where, OR_1_ and OR_2_ refer to the log odds ratio of the enrichment in conditions 1 and 2, respectively, while z refers to the z-score of the difference in log ORs. We then calculated the associated *p*-value from the z-score using the cumulative distribution function for the standard normal distribution. For each GO term type (i.e., Biological Process (BP), Cellular Component (CC), and Molecular Function (MF)), we adjusted these *p*-values using the Benjamini-Hochberg false discovery rate (FDR) method. We chose the top 5 terms in each group with FDR < 0.05 for visualization.

Next, we used the MEGENA R package (version 1.3) [6] to first build a planar filtered network (PFN) from significantly differentially correlated gene pairs (q < 0.05) and then identify multiscale gene modules in the PFN. We used the absolute value of the z-score for the difference in correlation between conditions as the network weights, and after calculating the perfuse forced network, we also normalized these weights, to follow the convention that network weights fall between 0 and 1. Next, we identified modules and hubs, using the default MEGENA parameters values, including module significance threshold of *p* < 0.05, a hub detection significance threshold of *p* < 0.05, 100 network permutations, and a module size of between 50 and 800. We then calculated the odds ratios of the enrichment of correlation class edges in each module using the hypergeometric test, and adjusted the enrichment *p*-values for each correlation class using the Benjamani-Hochberg method. Further, we performed the enrichment of GO terms in each modules using the GOstats R package [42] and org.Hs.eg.db GO annotation R package and adjusted the resulting *p*-values for all GO terms in each module with the Benjamini-Hochberg method. We visualized two of the networks derived from differential correlation modules using Cytoscape and created interactive versions of them via CyNetShare.

### Comparison to alternative module detection approaches

We sought to compare DGCA/MEGENA to two approaches to differential correlation module detection, DiffCoEx [44] and DICER [10]. For DiffCoEx, we downloaded the R script that the authors released in their Supplementary materials and used the same method and set of R commands they used therein. For DICER, we downloaded the Java executable file from the author’s website (http://acgt.cs.tau.ac.il/dicer/) on 8/21/2016 and ran it using Java v. 1.8.0_66. We designed a simulation study to assess the performance of different approaches for detecting differentially correlated modules. This simulation study uses many of the same parameters as our previous simulation study of differentially correlated gene pairs but has several unique features. First, instead of individual gene pairs that vary in correlation between conditions, we designated two modules of 30 genes each, a fraction of the pairs of which were positively correlated in one of the conditions but had no correlation in the other. In the simulation, we changed the fraction of positively correlated gene pairs in a module, defined as the network connectivity κ, from κ = 0.5 to 1, in increments of 0.1. Further, to ensure positive-definite covariance matrices, we set the numerical tolerance to 0.4 in simulating each of the multivariate normally distributed gene expression matrices. Because this numerical tolerance moderated the strength of the correlation difference between conditions, we increased the strength of correlated gene pairs to 0.9 in the positively correlated condition. Notably, we used the default 10 permutations and q-value threshold of 0.05 for DGCA in the simulation study to identify differentially correlated gene pairs as input to MEGENA for module detection. To be consistent with DICER, the minimum module size was set to 15 for both MEGENA and DiffCoEx. For each of the 10 simulation runs, we computed the highest sensitivity (i.e., the size of the detected module intersection with the true module) for all modules with less than 50 members for each module detection method. In each simulation run, we also computed the highest Jaccard index (i.e., the size of the intersection of the detected and modules divided by the union) for all modules for each module detection method. In the case that no modules were detected by a method, a pseudo-module comprised of all the genes in the simulation study was assigned, leading to a Jaccard index of 0.05 (30/600). We then compared the sensitivity statistics and Jaccard indices of all simulation runs between the methods at each network connectivity fraction and number of samples using a *t*-test.

We next compared the module detection methods in terms of their performance on the same set of filtered genes for ER+ and TN breast cancer RNA expression data as used by the DGCA/MEGENA pipeline. We first assess the relevance of the sets of modules identified by the three methods through the enrichment test for five gene signatures characterizing these breast cancer subtypes under study. The modules from each method were filtered by size to retain modules with greater than 25 and less than 1000 members. The five gene sets used were a set of genes associated with ER+ breast cancer in multiple data sets [45], a set of genes associated with TNBC in multiple data sets [46], a set of genes associated with ER signaling curated from several data sets [37, 38, 47–52], KEGG Cell Cycle genes, and KEGG DNA Mismatch Repair genes [47] (Additional file 1). The Benjamini-Hochberg adjustment method was used to correct the enrichment *p*-values for multiple testing, and we used an FDR threshold of 0.3 for all the enrichments of the gene sets. We then tested how the modules detected by one method are conserved in each other method based on a significance threshold (*p* < 0.05) of the Benjamini-Hochberg adjusted Fisher’s Exact Test (FET) enrichment *p*-value. A module is defined as unique to a method if it does not significantly overlap with any module identified by any other method. This procedure allowed us to determine the proportion of modules specific to each of the three methods or in any pairwise comparison of modules.

## Results

### Simulation study to measure the accuracy of detecting differential correlation

**Fig. 4:**
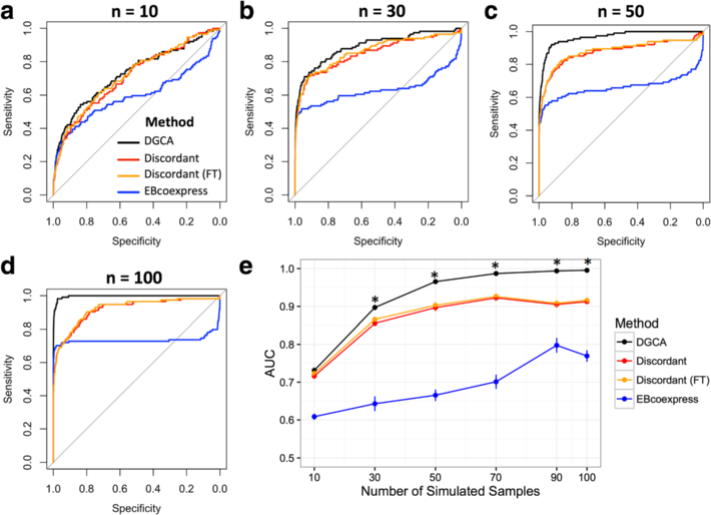
Comparing DGCA to alternatives in the differential correlation simulation study. **a-d**: Representative receiver operating characteristic (ROC) curves show the ability of DGCA, EBcoexpress, and Discordant with and without the use of the Fisher z-transformation (FT) to accurately detect truly differential correlated gene pairs at different simulated sample sizes in each condition, including *n* = 10 **a**, *n* = 30 **b**, *n* = 50 **c**, and *n* = 100 **d**. Black lines show the ROC curves of DGCA, while blue lines show the ROC curves of EBcoexpress, red lines show Discordant without the Fisher transformation, and orange lines show Discordant with the Fisher transformation. **e**: Comparison of area under curve (AUC) statistics for 5 runs of the simulation study using each of the methods at different numbers of samples, where errors bars represent the standard error of the mean. Asterisks indicate a Bonferroni-adjusted significant difference in the AUCs (*p* < 0.0083) between DGCA and each of the other methods, tested using a two-sided *t*-test

**Fig. 5:**
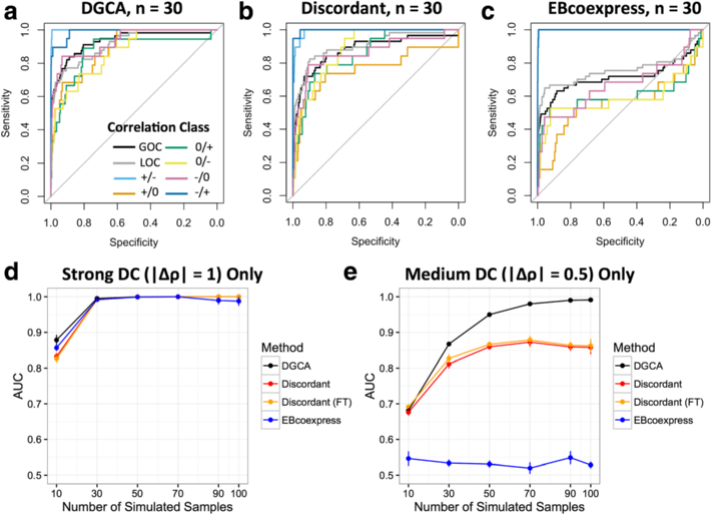
Comparing DGCA to alternatives segregated by the strength of correlation difference. **a**-**c**: Representative receiver operating characteristic (ROC) curves show the ability of DGCA **a**, EBcoexpress **b**, and Discordant (with the use of the Fisher z-transformation) **c** to accurately detect truly differential correlated gene pairs in each of the six differential correlation classes that specify a difference in correlations between conditions, as well as when comparing all gene pairs with a gain in correlation (GOC) or a loss of correlation (LOC) in one condition compared to the other, at a sample size of *n* = 30. **d**-**e**: Comparison of area under curve (AUC) statistics for 5 runs of the simulation study using each of the three methods at different numbers of samples, where errors bars represent the standard error of the mean, segregated to only those gene pairs with a strong difference in correlation (absolute value of difference in ρ = 1; **d**) or segregated to only those gene pairs with a medium strength difference in correlation (absolute value of difference in ρ = 0.5; **e**). Asterisks indicate a Bonferroni-adjusted significant difference in the AUCs (*p* < 0.0083) between DGCA and each of the other methods, tested using a two-sided *t*-test

DGCA is an R package designed to detect differences in the correlations of gene pairs between distinct biological conditions. We first developed a simulation study to confirm that DGCA can accurately detect differential correlation relationships between gene pairs. Specifically, we designed an *in silico* experiment with 600 genes and 114 differentially correlated gene pairs out of 179,700 total gene pairs. Thirty eight of the truly differentially correlated gene pairs had a strong difference (1) in correlation coefficient between conditions, while 76 had a medium difference (0.5) in correlation coefficient between conditions. The accuracy of DGCA increased as the number of simulated gene expression samples in each condition increased (Fig. 4a-d). DGCA also demonstrated significantly higher accuracy under these simulation conditions than Discordant with and without use of the Fisher z-transformation for *n* = 30, 50, 70, 90, and 100 samples (two-sided Student’s *t*-test, *p*-value < 0.0083), although the two packages did not significantly differ in accuracy at *n* = 10 samples (Fig. 4e). DGCA demonstrated significantly higher accuracy in the simulation study than EBcoexpress at all of the sample sizes tested (Fig. 4e). Furthermore, DGCA was substantially faster than the other two methods (Table 1). At *n* = 30 samples, all three methods more accurately detected gene pairs with strong difference in correlation than gene pairs with medium difference in correlation, but there were no major differences within methods between correlation classes within the strong or medium groups (Fig. 5a-c). All the methods have similar power to detect gene pairs with strong differences in correlation at any of the sample sizes tested (Fig. 5d). For medium strength gene pairs, DGCA does not significantly differ from Discordant at *n* = 10 and 30 in terms of accuracy, but outperforms Discordant at *n* = 50, 70, 90, and 100, while DGCA outperforms EBcoexpress at all sample sizes tested (Fig. 5e). Therefore, DGCA outperforms the two established approaches, EBcoexpress and Discordant, in our simulation study.

**Table 1:** Speed measurements for DGCA, Discordant, and EBcoexpress across in the simulation study

Number of Simulated Samples	DGCA Execution Time (s)	DGCA (10 Perm.) Execution Time (s)	Discordant Execution Time (s)	EBcoexpress Execution Time (s)
10	2.4 +/- 0.2	18.2 +/- 0.2	259.1 +/- 33.1	2625.5 +/- 271.8
30	2.6 +/- 0.2	17.4 +/- 0.2	293.5 +/- 17.5	282.6 +/- 34
50	1.9 +/- 0.2	16 +/- 0.7	266.3 +/- 28.3	260.9 +/- 36.9
70	2 +/- 0.1	17.1 +/- 0.3	240.4 +/- 1.3	222.4 +/- 2
90	1.9 +/- 0.1	18.6 +/- 0.3	210.3 +/- 17.3	1983.9 +/- 126.2
100	2.1 +/- 0.2	15.9 +/- 0.7	246.8 +/- 34.5	2479.1 +/- 380.8

The time (in seconds) for each analysis tool to take an input gene expression data with 600 genes and the given number of samples and output a table of differential correlation predictions for all 179,700 gene pairs. For DGCA, speed times both with and without 10 permutations (the default number of permutations in DGCA) are shown. These numbers were calculated from 5 runs of the simulation study, representing the arithmetic mean +/- the standard error of the mean across the simulations

### Differential correlation of p53 targets in normal and p53-mutated breast cancer tumor samples

**Fig. 6:**
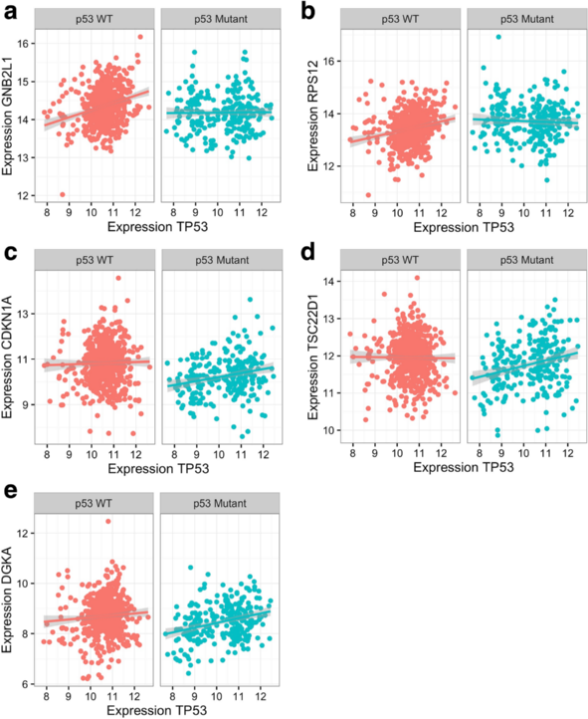
Expression correlations of five genes with *TP53* in samples with and without p53 mutations. Expression values for genes from breast cancer samples without p53 mutation, or p53 wildtype (WT; red, *n* = 590), compared to samples with non-silent p53 coding mutations (blue, *n* = 254). For each gene pair, the expression of TP53 is on the x-axis, while the expression of *GNB2L1* **a**, *RPS12* **b**, *CDKN1A* **c**, *TSC22D1* **d**, and *DGKA* **e** are on the y-axis. For visualization purposes, a linear model was fit to the data in each comparison, with the grey lines representing 95% confidence intervals. This visualization was made using a DGCA wrapper function to the ggplot2 R package

We downloaded RNA-seq data of breast cancer samples with and without non-silent p53 DNA mutations from TCGA (*n* = 590 non p53-mutated samples, *n* = 254 non-mutated samples), and corrected the data for known covariates. We chose this data set because the ability of p53 to affect the expression of its downstream targets is known to be altered following p53 mutation [53, 54]. We filtered the RNA-seq data to select 295 genes in the data set as the p53 signature that have previously been associated with p53 [36–38]. Since p53 is known to be a transcriptional activator, we restricted our analysis to positive correlations with *TP53* in the two groups (see Methods). We then quantified the significance of these differential correlation relationships using permutation testing. We identified five genes that have a significant (q < 0.05; corresponding to nominal *p*-value 0.0098) change in correlation with *TP53* in the group of samples with p53 mutations (Fig. 6; Table 2; Additional file 2). Two of these are due to a loss of correlation following p53 mutation (*GNB2L1*, *RPS12*), while three are due to a gain of correlation (*TSC22D1*, *CDKN1A*, *DGKA*). The gene with the strongest loss of correlation with *TP53* is *GNB2L1* (a.k.a. *RACK1*), a ribosomal gene whose expression is strongly predictive of breast cancer outcome [55]. *CDKN1A*, a well-established transcriptional target of p53 that encodes the protein p21, demonstrated an increased correlation with *TP53* in the group of samples with p53 mutations, possibly reflecting compensatory p53-independent transcriptional activation of *CDKN1A* [56].

**Table 2:** Differential correlations of genes with *TP53* between non p53-mutated and p53-mutated samples

Gene	p53 WT Cor.	p53 Mut. Cor.	z-score	Empirical *p*-Value	q-Value	Classes
GNB2L1	0.257	0.01	-3.25	3.81E-05	0.008	+/0
RPS12	0.231	-0.002	-3.02	0.0001	0.009	+/0
TSC22D1	-0.027	0.227	2.97	0.0001	0.009	0/+
CDKN1A	0.019	0.227	2.73	0.0004	0.02	0/+
DGKA	0.057	0.252	2.58	0.0007	0.03	0/+
CARM1	0.172	-0.036	-2.24	0.0025	0.091	NonSig
DDB2	0.025	0.191	2.16	0.0033	0.094	NonSig
TNFRSF10B	0.194	0.034	-2.09	0.0043	0.094	NonSig
RPL18	0.158	-0.01	-2.05	0.0049	0.094	NonSig
ATF3	0.004	0.161	2.04	0.0051	0.094	NonSig
PYCARD	0.205	0.05	-2.04	0.0051	0.094	NonSig
TAX1BP3	0.305	0.156	-2.03	0.0052	0.094	NonSig
EI24	0.154	-0.007	-1.99	0.0059	0.097	NonSig
TNFRSF10C	0.179	0.03	-1.94	0.0069	0.097	NonSig
CCNG1	0.149	-0.092	-1.93	0.0072	0.097	NonSig
PML	0.016	0.163	1.92	0.0076	0.097	NonSig
CREBBP	0.153	0.006	-1.91	0.0077	0.097	NonSig
ABAT	0.145	-0.037	-1.88	0.0085	0.102	NonSig
NOL8	0.228	0.089	-1.85	0.0094	0.107	NonSig

The output of running DGCA on the p53 pathway gene set in the breast cancer RNA-seq samples, comparing correlations of these genes with *TP53* in p53-wildtype samples to correlations in p53-mutated samples, using the option to consider only positive correlations in calculating differential correlation between conditions. The top 20 gene pairs are shown here, while the rest are available in the Supplementary data. The “Classes” column indicates the correlation class of each of the gene pairs where, e.g., “+/0” indicates a significant (q < 0.05) positive correlation in the p53-wildtype samples and no significant correlation in the p53-mutated samples. Note that the significance for the correlations within each condition is not adjusted for multiple comparisons. 10,000 permutation samples were generated in order to estimate empirical *p*-values, using a pooled reference distribution approach, from which q-values were calculated

*WT* Wildtype, *Mut.* Non-silent p53 mutation

**Fig. 7:**
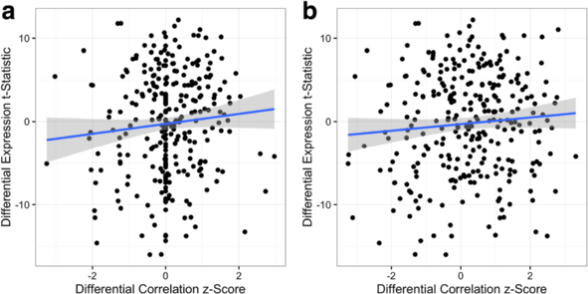
Comparing differential expression and differential correlation with *TP53* in samples with and without p53 mutations. For each gene, we plot both DGCA’s calculated differential correlation z-score between that gene and *TP53* in p53 non-mutated breast cancer samples and p53-mutated samples (x-axis), as well as limma’s differential expression t statistic for that gene’s differential expression between the same p53 wildtype samples and p53-mutated samples (y-axis). When differential correlation z-scores are calculated on positive correlation values only **a**, the Spearman correlation between these two measures is not significant (ρ = 0.08, *p*-value = 0.15), and when differential correlation z-scores are calculated across all correlation values **b**, the Spearman correlation between these two measures is also not significant (ρ = 0.06, *p*-value = 0.30). The blue line represents a linear model of the best fit, with the grey lines representing 95% confidence intervals, computed using ggplot2

We further explored the relationship between differential correlation and differential expression. We performed differential expression analysis on these genes using the R package limma (Additional file 3). We then calculated the Spearman correlation between the magnitude of differential expression (measured by the t-statistic) and the differences in positive correlation values with *TP53* for all of the genes. Although these two measures trended in the same direction, they had no significant correlation between them (ρ = 0.08, *p*-value = 0.15; Fig. 7a). Because this lack of correspondence may have been influenced by our restriction to differences in positive correlations, we also measured the correlation between differential expression and differential correlation with *TP53* across all the correlations, and found no correlation in that case either (ρ = 0.06, *p*-value = 0.30; Fig. 7b). The lack of correlation corroborates our theoretical claim that differential expression and differential correlation are complementary approaches to identifying differences in gene expression between conditions.

**Fig. 8:**
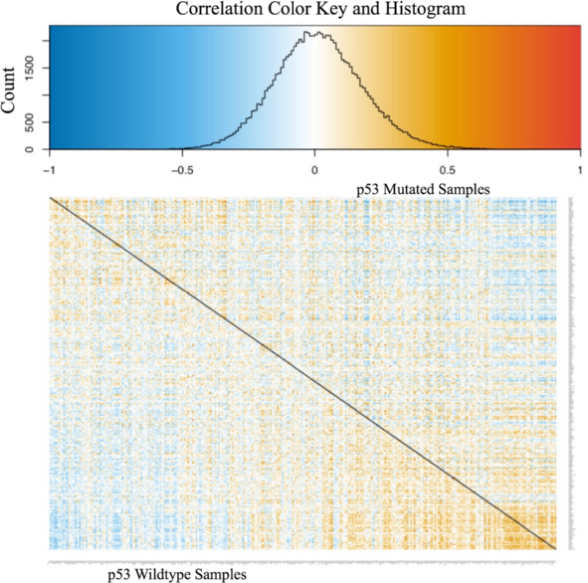
Differential correlations between the genes in the p53 pathway in p53 non-mutated and p53-mutated breast cancer samples. The top panel shows a histogram of the correlation values pooled between both conditions that also demonstrates the color schema used to indicate the expression correlation strength for each gene pairs. The bottom panel shows a heatmap of correlation values in the two conditions. The bottom left panel shows the correlation of gene pairs in the non-p53-mutated breast cancer RNA-seq samples, while the upper right panel shows the correlation of gene pairs in the samples with (non-silent) p53 mutations. The diagonal is marked black as each gene’s self-correlations from both conditions are omitted. Genes are ordered by the median difference of z-score in correlation between that gene and all of the other genes present in both the rows and columns. This plot was made using the DGCA wrapper function to the gplots R package

To gain more insights into the overall change in correlation structure in RNA expression matrices between p53-mutated and p53-non-mutated breast cancer samples, we used DGCA to visualize the all gene pair correlations in both conditions (Fig. 8). Genes in this heatmap are ordered by their median z-score correlation difference with all of the other genes in the filtered set between the two conditions, without the restriction to positive correlations. When we quantified the difference in correlation between all gene pairs using permutation testing, we identified a global loss of correlation between p53-mutated and p53-wildtype samples (median difference in z-score (dz) = -0.06, *p* = 0.007), suggesting that p53 mutations decrease the correlations among the p53 signature genes. Because visual inspection of the heatmap indicated that a subset of genes tended towards gain in correlation while others tended towards a loss in correlation, we used permutation testing to measure whether each gene’s median change in correlation was more extreme than expected, and adjusted the resulting empirical *p*-values for multiple hypothesis testing. Using this approach, we identified genes with significant changes in median correlation in p53-mutated samples (Table 3; Additional file 4). Notably, the transcription factor *FOXA1* has a gain in correlation with the other genes in the p53 signature in the p53-mutated breast cancer samples (median dz = 0.48, *p* < 1e-4). This suggests that *FOXA1* may play a role in transcriptional compensation following p53 mutation, which is consistent with the finding that *FOXA1* binding sites are enriched in p53 binding sites [57]. On the other hand, the transcription factor *ATF3* has a loss of correlation with the other genes in the set (median dz = -0.67, *p* < 1e-4), which is consistent with its highly synergistic role with p53 in mediating transcription [58] and ability to directly bind to and suppress mutant p53 [59]. *LDHB* also has a strong loss of correlation in p53-mutated samples (median dz = -0.56, *p* < 1e-4). Since *LDHB* (lactate dehydrogenase B) is a marker of highly glycolytic cancers [60], its broad dysregulation following p53 mutation may reflect the disrupted role of p53 in regulating glycolytic activity [61]. These examples highlight the ability of DGCA to parse out the individual components of the differential correlation structure between conditions.

**Table 3:** Median differences in correlation among p53 pathway genes between non p53-mutated and p53-mutated samples

Gene	Median z-Score Difference	Empirical *p*-Value	Adjusted *p*-Value
SERPINB5	-0.730683588	*p* < 1e-4	*p* < 1e-4
ATF3	-0.666702829	*p* < 1e-4	*p* < 1e-4
FAS	-0.590308897	*p* < 1e-4	*p* < 1e-4
ARID3A	0.584112447	*p* < 1e-4	*p* < 1e-4
RPS12	-0.565836003	*p* < 1e-4	*p* < 1e-4
SPHK1	-0.56379232	*p* < 1e-4	*p* < 1e-4
LDHB	-0.562303813	*p* < 1e-4	*p* < 1e-4
MET	-0.527886541	*p* < 1e-4	*p* < 1e-4
CX3CL1	-0.511204357	*p* < 1e-4	*p* < 1e-4
KRT17	-0.504964779	*p* < 1e-4	*p* < 1e-4
FOXA1	0.482088394	*p* < 1e-4	*p* < 1e-4
IGFBP3	-0.438982138	*p* < 1e-4	*p* < 1e-4
MAP4K4	-0.436176474	*p* < 1e-4	*p* < 1e-4
NOTCH1	-0.610467618	0.001	0.013409091
PMS2	0.535342391	0.001	0.013409091
MLH1	0.52374045	0.001	0.013409091
EGFR	-0.515311146	0.001	0.013409091
FOXO3	-0.499303329	0.001	0.013409091
PPP1R15A	-0.487186159	0.001	0.013409091
TADA2B	0.480724824	0.001	0.013409091

The top 20 genes by their median difference in correlation for each gene and all other genes in the p53 pathway gene set between non p53-mutated and p53-mutated breast cancer RNA-seq samples. 1000 permutation samples were generated in order to estimate empirical *p*-values for each gene, which were then adjusted by the Benjamini-Hochberg method to control the false discovery rate among this set of genes

### Differential correlation of PTEN targets in normal and PTEN-mutated breast cancer tumor samples

**Fig. 9:**
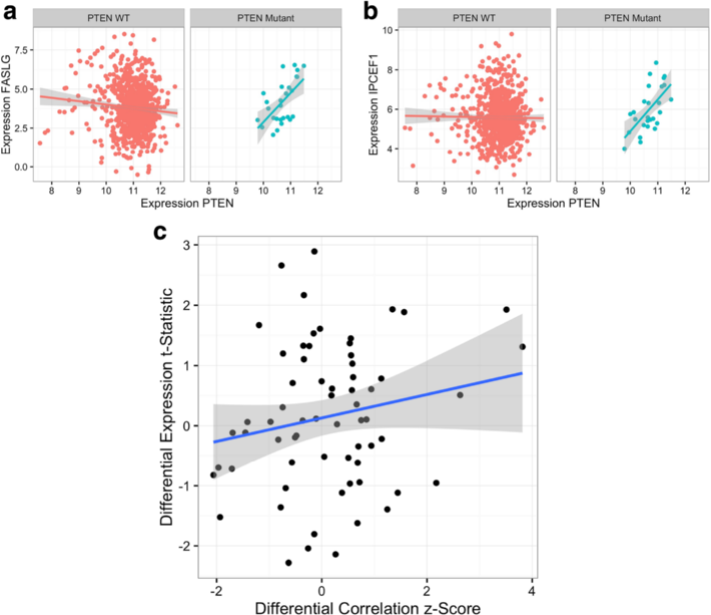
Summary of differential correlation results of PTEN in samples with and without PTEN mutations. **a**-**b**: Expression values for genes from breast cancer samples without PTEN mutation, or PTEN wildtype (WT; red, *n* = 816), compared to samples with non-silent PTEN mutations (blue, *n* = 27). For each gene pair, the expression of *PTEN* is on the x-axis, while the expression of *FASLG* **a** and *IPCEF1* **b** are on the y-axis. For visualization purposes, a linear model was fit to the data in each comparison, with the grey lines representing 95% confidence intervals. **c**: For each gene, we plot both DGCA’s calculated differential correlation z-score between that gene and *PTEN* in PTEN non-mutated breast cancer samples and PTEN-mutated samples (x-axis), as well as limma’s differential expression t statistic for that gene’s differential expression between the same PTEN non-mutated samples and PTEN-mutated samples (y-axis). The correlation between the two measures is not significant (ρ = 0.13, *p* = 0.29). The blue line represents a linear model of the best fit, with the grey lines representing 95% confidence intervals

We used a similar approach to measure the differential correlation of genes known to be associated with *PTEN* following PTEN DNA mutation, using the same processed breast cancer RNA expression data as we used for p53. PTEN is typically considered a tumor suppressor, and its role in breast cancer pathogenesis has been suggested to be related to loss of PTEN protein activity [62]. We filtered the RNA-seq data to select 66 genes in the data set as the PTEN signature that have previously been associated with PTEN [39–41]. Because PTEN was not considered as a transcriptional activator, we did not restrict the correlations to positive values prior to calculating differential correlations as we did for p53 (see Methods). We identified two genes with a significant gain in correlation with *PTEN* in breast cancer samples with PTEN mutations (*n* = 27) compared to samples without PTEN mutations (*n* = 816), *FASLG* and *IPCEF1* (Table 4, Fig. 9a-b, Additional file 5). *FASLG* encodes Fas ligand, which induces apoptosis upon binding to Fas, and polymorphisms in which contribute to breast cancer risk [63]. Fas signaling typically activates PTEN as a part of its promotion of apoptosis [64]. The expression of Fas and PTEN have previously been found to be negatively correlated in prostate cancer [65], consistent with the negative correlation we identify between *PTEN* and *FASLG* in the PTEN wildtype breast cancer samples, suggestive of negative regulation at baseline. In the absence of PTEN protein activity, activation of Fas may lead to compensatory increased transcription of *PTEN* in a failed attempt of negative regulation, perhaps mediated in part by microRNA-21 [66], thus inducing a strong positive correlation between the expression of these two genes. *IPCEF1* encodes a scaffold protein involved in signal transduction downstream of growth factors and Ras [67]. *IPCEF1* is less well-studied, so its differential correlation with *PTEN* in the presence of PTEN mutations may provide a way to study its function. We next examined the relationship between differential expression and differential correlation with respect to PTEN. As in the case of p53, there is no correlation between the genes differentially expressed between the groups with and without PTEN mutations and the genes differentially correlated with *PTEN* (ρ = 0.13, *p* = 0.29; Fig. 9c; Additional file 6).

**Table 4:** Differential correlations of genes with *PTEN* between non PTEN-mutated and PTEN-mutated samples

Gene	PTEN WT Cor.	PTEN Mut. Cor.	z-score	Empirical *p*-Value	q-Value	Classes
FASLG	-0.076	0.628	3.816	0.0003	0.015	-/+
IPCEF1	-0.012	0.627	3.511	0.0008	0.021	0/+
PIK3CD	0.015	0.52	2.63	0.01	0.169	NonSig
FBXW7	0.0001	0.433	2.177	0.031	0.381	NonSig
STUB1	-0.075	-0.473	-2.06	0.041	0.381	NonSig
WWP2	-0.107	-0.482	-1.965	0.051	0.381	NonSig
MAPK3	0.013	-0.379	-1.934	0.054	0.381	NonSig
AKT1	0.077	-0.28	-1.71	0.087	0.492	NonSig
BCAR1	-0.025	-0.369	-1.696	0.09	0.492	NonSig
NCOA3	-0.055	0.272	1.566	0.116	0.573	NonSig
PARK7	-0.001	-0.301	-1.453	0.144	0.587	NonSig
CBL	0.069	0.36	1.444	0.147	0.587	NonSig
MVP	0.215	-0.082	-1.414	0.155	0.587	NonSig
SOS1	0.022	0.299	1.343	0.176	0.62	NonSig
ANAPC5	-0.068	0.195	1.246	0.209	0.65	NonSig
PIK3R1	0.309	0.065	-1.193	0.229	0.65	NonSig
RBL2	0.109	0.338	1.139	0.251	0.65	NonSig
CDC27	0.002	0.239	1.131	0.254	0.65	NonSig
SLC9A3R1	-0.065	-0.267	-0.975	0.323	0.65	NonSig

The output of running DGCA on the PTEN pathway gene set in the breast cancer RNA-seq samples, comparing correlations of these genes with *PTEN* in PTEN-wildtype samples to correlations in PTEN-mutated samples. The top 20 gene pairs are shown here, while the rest are available in the Supplementary data. The “Classes” column indicates the correlation class of each of the gene pairs. Note that the significance for the correlations within each condition is not adjusted for multiple comparisons. 10,000 permutation samples were generated in order to estimate empirical *p*-values, using a pooled reference distribution approach, from which q-values were calculated

*WT* Wildtype, *Mut.* Non-silent PTEN mutation

### Global differential correlations between estrogen receptor-positive and triple-negative breast cancer samples

**Fig. 10:**
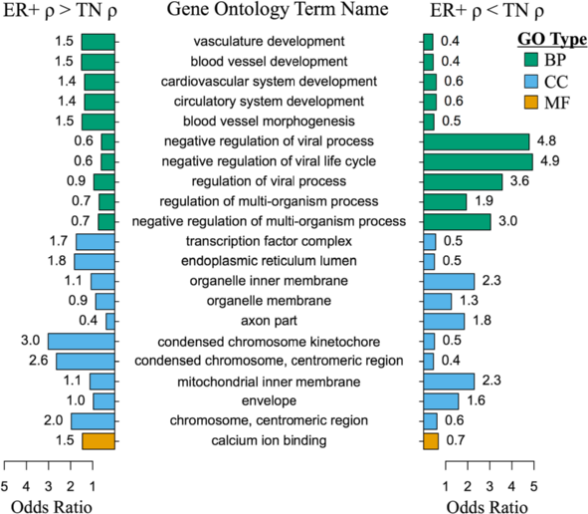
Gene ontology enrichment of genes associated with gain or loss of correlation in ER+ samples compared to TN. Genes identified as members of gene pairs with a significant gain in correlation (q < 0.01) in the estrogen receptor-positive (ER+) samples compared to the triple negative (TN) breast cancer samples were used as inputs to gene ontology (GO) enrichment analysis in GOstats. GO terms with less than 50 or greater than 600 gene symbol members were filtered out included for the purposes of interpretability. Odds ratios for the GO enrichment of terms were compared between these two groups, and *p*-values were adjusted using the Benjamini-Hochberg method for each GO term category to yield false discovery rates (FDR). Up to 5 GO terms enriched in one of the groups compared to the other at FDR < 0.3 are shown. No terms from the “Molecular Function” GO term category portion passed this FDR threshold and are therefore not shown. This plot was made using the DGCA wrapper function to the plotrix R package

We next sought to use DGCA to make a global comparison of gene expression correlation patterns between estrogen receptor-positive (ER+) and triple-negative (TN) breast cancers in TCGA (*n* = 625 ER+ samples, *n* = 89 TN samples). We filtered out genes in the bottom 25^th^ percentile of median expression and/or dispersion index of expression, which yielded a total of 10,530 genes. We found the difference in Pearson correlations among all of these gene pairs across the two conditions, and identified 520,907 differentially correlated gene pairs at q-value < 0.05 (corresponding to nominal *p*-value < 3.1e-4) between the ER+ and TN breast cancer gene expression samples. We first measured gene ontology (GO) of the genes in gene pairs with a gain of correlation in ER+ samples to the GO enrichment of genes found in gene pairs with a gain correlation in TN samples, but found no significant differences in the GO term enrichments between groups, likely due to the small number of unique genes in each category (*n* = 49 genes found uniquely in gene pairs with a gain of correlation in ER+ samples and *n* = 45 corresponding genes for TN samples). However, by restricting to the 97,644 gene pairs at q < 0.01 (corresponding to nominal *p*-value < 7.3e-6) for specificity, we were able to perform GO enrichment on a larger set of genes unique to gene pairs with a gain of correlation in ER+ samples (*n* = 1201 genes) or a gain correlation in TN samples (*n* = 1320; Fig. 10). Genes in gene pairs with a gain of correlation in ER+ samples were enriched in the GO term vasculature development (ER+ OR = 1.5, FDR of difference with TN = 1.6e-4), which makes sense in light of the finding that ER+ breast cancer samples harbor a relatively high proportion of blood vessels in the tumor environment [68]. Further, genes in ER+ specific gene pairs were enriched in calcium ion binding (OR = 1.5, FDR = 0.008), which is consistent with the ability of calcium to stimulate estrogen receptor-alpha expression and mimic estrogen [69, 70]. On the other hand, genes in gene pairs with a gain of correlation in TN samples were enriched in the GO term negative regulation of viral life cycle (TN OR = 4.9, FDR = 0.006), which may be explained by reports that a subset of TN breast cancers are associated with viral infection [71]. Surprisingly, despite the presence of multiple gene sets related to estrogen-regulated genes in the GO annotation set employed, including “estrogen receptor activity” and “estrogen receptor binding”, none of these were identified as significantly enriched in differentially correlated gene pairs unique to ER+ or TN samples. This may be because changes in the correlations of ER-responsive genes with other genes occur in both ER+ and TN samples, suggesting that DGCA can pull out novel biology for further investigation.

**Fig. 11:**
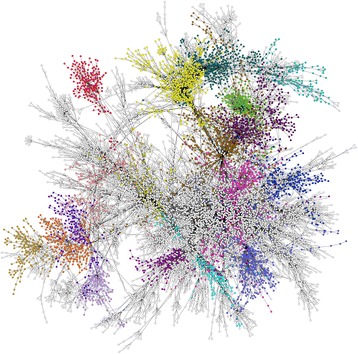
Planar filtered network of differentially correlated genes. Gene pairs with a significant change in correlation (q < 0.05) in estrogen receptor-positive (ER+) compared to triple negative (TN) breast cancer samples were to construct a planar filtered network shown here. The modules identified at *p* < 0.05 in this network with between 100 and 800 members are identified with distinct colors. This plot was made using Cytoscape (version 3.2.1)

**Fig. 12:**
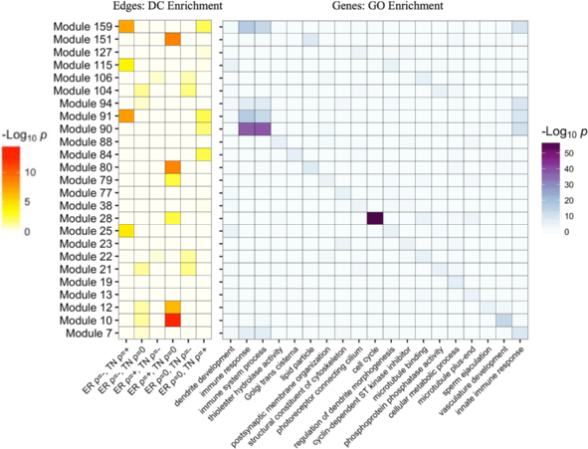
Enrichment of GO terms and differential correlation classes in the multiscale modules. This plot displays the enrichments of each of the multiscale modules (whose sizes are between 100 and 800 members) in the ER+ vs TN differential correlation network. The left panel shows the enrichment (Benjamini-Hochberg adjusted -log_10_ *p*-value) of edges in each of the differential correlation (DC) classes in each of the modules. The right panel shows the most significantly enriched gene ontology (GO) term (-Benjamini-Hochberg adjusted log_10_ *p*-value) for the genes in each of the corresponding modules, along with the GO enrichment of that same GO term in all the other modules. This plot was made using a DGCA wrapper function to R package ggplot2

**Fig. 13:**
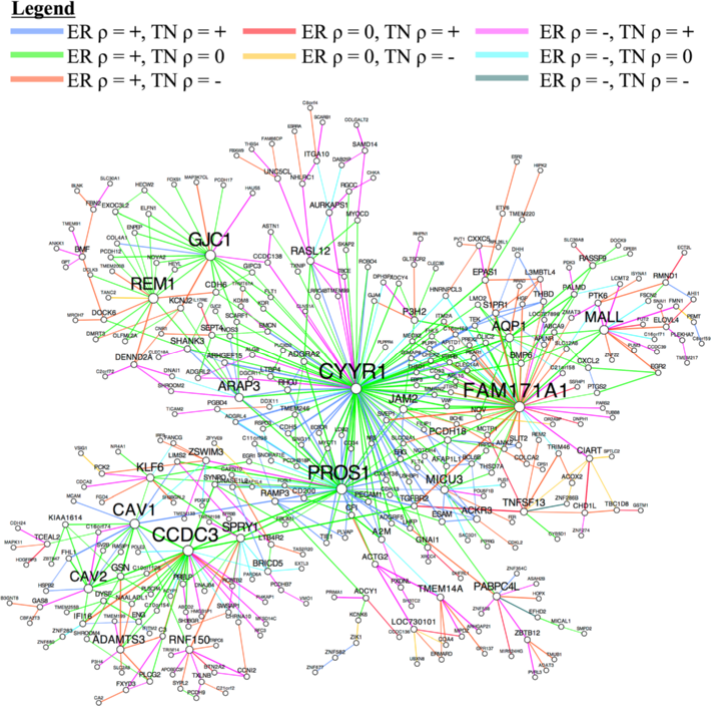
The differential correlation module most enriched in gene pairs that gain in correlation in ER+ breast cancer. This module of genes was identified using MEGENA and chosen for downstream analysis because it was most enriched for the gene pairs with positive correlations in ER+ breast cancer but no significant correlation in triple negative (TN) breast cancer. Node size and gene symbol text size are proportional to the number of connections for each gene. Edges are colored according to the differential correlation class (see Legend), while edge weight is proportional to the absolute value of the z-score for the difference of correlation between ER+ and TN breast cancer samples. This plot was made using Cytoscape (version 3.2.1)

**Fig. 14:**
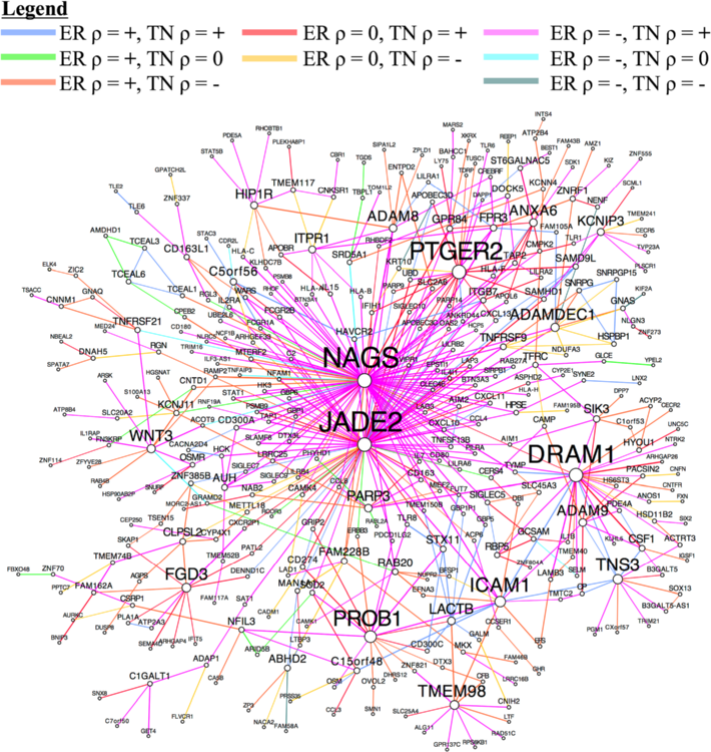
The differential correlation module most enriched for the gene pairs that gain correlation in TN breast cancer. This module of genes was identified using MEGENA and chosen for downstream analysis because it was most enriched for the gene pairs that were positively correlated in TN breast cancer but were either negatively or not significantly correlated in ER+ breast cancer. Node size and gene symbol text size are proportional to the number of connections for each gene. Edges are colored according to the differential correlation class (see Legend), while edge weight is proportional to the absolute value of the z-score for the difference of correlation for that gene pair between ER+ and TN breast cancer samples. This plot was made using Cytoscape (version 3.2.1)

In order to identify coherent subnetworks and hub genes constituted by differential correlations, we integrated DGCA with MEGENA (Multiscale Embedded Gene Co-expression Network Analysis) [6] and used the set of 520,907 gene pairs found at q < 0.05 as inputs to Planar Filtered Network (PFN) construction. MEGENA projects candidate interactions onto a topological sphere and parses the resulting planar filtered network into multiscale modules (subnetworks) defined at multiple resolutions, and has been shown to effectively reconstruct gene regulatory networks [6]. The resulting planar filtered network had 16,737 edges, from which we identified 25 modules using multiscale clustering analysis (Fig. 11). We measured the enrichment of the edges in these modules in each of the differential correlation classes, and further found their most enriched GO terms (Fig. 12). We first focused on the module with the strongest enrichment of edges with positive correlation in the ER+ samples and no correlation in the TN samples (Module 11; OR = 2.5, *p* = 4.2e-7), which is most enriched in the GO term vasculature development (OR = 4.2, *p* = 1.3e-13). We also used MEGENA to identify hub genes (at FDR < 0.05) in this module, which revealed that *CYYR1*, *FAM171A1*, *PROS1*, *CCDC3*, *GJC1*, *REM1*, and *CAV1* are its hub genes (Fig. 13). The locus at *CYYR1* encodes seven or more *CYYR1* alternatively spliced isoforms as well as an antisense gene with high expression variability across tissue types [72]. Our data suggests that *CYYR1* drives and/or is associated with a strong reprogramming of transcription in ER+ compared to TN breast cancer samples. We next focused on the module with the strongest enrichment of edges with negative correlation in ER+ samples and positive correlation in TN samples (Module 13, OR = 2.3, *p* = 2.4e-8), which is also significantly enriched in edges with no correlation in ER+ samples and positive correlation in TN samples (OR = 1.7, *p* = 0.005). This module is most enriched for the GO term immune response (OR = 4.6, *p* = 7.9e-17), consistent with reports of the relatively strong role of the immune system in mediating TNBC [73–75]. The hub genes identified in this network are *NAGS*, *JADE2*, *DRAM1*, *PTGER2*, and *PROB1* (Fig. 14). Notably, the protein product of *JADE2* has been found to act as a ubiquitin-ligase to regulate the activity of the histone demethylase LSD1 in neuronal differentiation [76], suggesting that it may play a role in shifting the immune-related epigenetic landscape in TNBC samples. Interactive figures for these two differential correlation networks are available online (see “Availability and requirements”).

### Evaluation of differential correlation module detection approaches

**Fig. 15:**
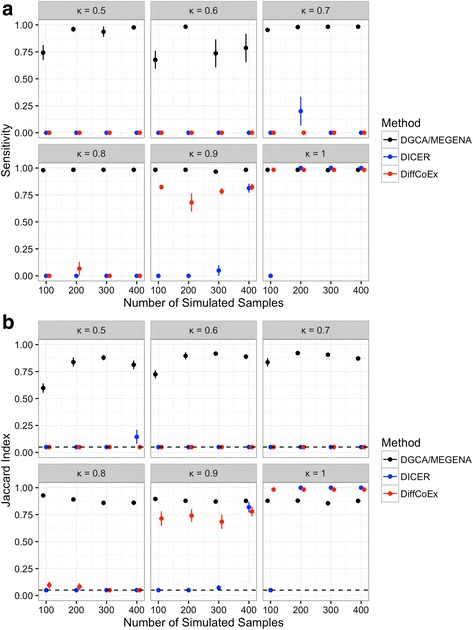
Performance comparison of DGCA/MEGENA, DICER and DiffCoEx in detecting differential correlation modules. In the simulation study, there were two modules and 30 genes were assigned to each module, within which a fraction (κ) of the gene pairs had differential correlation, where κ varied from 0.5 to 1 across simulation runs. We also simulated different sample sizes in each condition (*n* = 100, 200, 300, and 400). The highest sensitivity (**a**; dots = means, lines = standard errors of the mean) and Jaccard index **b** between the detected and true modules were calculated and averaged for each method (black for DGCA/MEGENA, blue for DICER and red for DiffCoEx) across all the independent runs in the simulation study. In the case that no module was detected by a method, all the genes were considered to belong to one single module for calculating Jaccard index, indicated by the dotted horizontal line. Each Jaccard index mean was calculated from ten simulation runs and two true modules per run

To evaluate the performance of DGCA/MEGENA in detecting differential correlations, we first compared it with two established methods, DiffCoEx [44] and DICER [10], using a simulation study. In this simulation, we designed two differential correlation modules of 30 genes each, out of a total of 600 genes. In each module, a fraction of gene pairs (ranging from κ = 0.5 to 1) have significant positive correlations in one condition but no significant correlation in the other. For each of the true modules across various numbers of simulated samples (*n* = 100 to 400), we identified the top sensitivity and Jaccard index statistics of the modules detected by each method and then compared them (Fig. 15). DGCA/MEGENA identified modules with significantly higher sensitivity and Jaccard indices than DiffCoEx and DICER in all the simulation conditions that have κ < 1 (t-tests, all unadjusted *p*-values < 0.05), with the exception of the Jaccard index comparison with DICER at κ = 0.9, *n* = 400 samples (*p* = 0.25). In the extreme condition where all the gene pairs are differentially correlated (κ = 1), DiffCoEx identifies modules with higher Jaccard indices (*p*-values < 0.05) but not higher sensitivities than DGCA/MEGENA at all sample sizes, while DICER identifies modules with higher sensitivities and Jaccard indices than both methods at *n* = 200, 300, and 400 (t-tests, all unadjusted *p*-values < 0.05). However, even under the extreme and limited condition of κ = 1, the practical performance difference between DGCA/MEGENA and the other two methods is very small. In contrast, DiffCoEx and DICER usually fail to identify the differentially correlated modules under the more general conditions (κ < 0.9). In summary, DGCA/MEGENA demonstrated the best performance in detecting differential correlation modules under the more general simulation conditions.

**Fig. 16:**
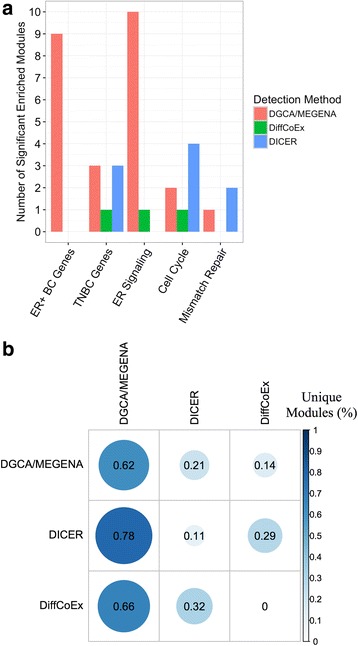
Comparison of modules detected with DGCA/MEGENA to those detected with DiffCoEx and DICER. **a**: The number of modules significantly enriched (Fisher’s Exact Test; FDR < 0.3) that were detected by each of DGCA/MEGENA (*red*), DiffCoEx (*green*), and DICER (*blue*) in five different gene sets selected for their relevance to breast cancer. **b**: The proportion of modules detected by each of the three methods that are not significantly enriched in (i.e., do not overlap with) any of the modules detected by one (off-diagonals) or both (diagonals) of the other methods, by Fisher’s Exact Test, Benjamini-Hochberg adjusted *p*-value < 0.05. Columns labels denote the reference method compared; e.g., 62% of DGCA/MEGENA-detected modules do not significantly overlap with modules detected by either method, and 78% of DGCA/MEGENA-detected modules do not significantly overlap with any DICER-detected modules

We next examined whether the differential correlation modules identified by DGCA/MEGENA on the ER+ and TN breast cancer RNA expression differed from those identified by DiffCoEx [44] and DICER [10] (Additional file 7). Although these alternative methods do not automatically specify gene-gene links or identify hub genes, they do also identify differentially correlated gene modules. We first counted the number of modules identified by each method that were significantly enriched (FDR < 0.3) in five gene sets chosen because of their importance in breast cancer (Fig. 16a; Additional file 1). We found that DGCA/MEGENA identified the most modules significantly enriched in genes associated with ER+ breast cancer and estrogen receptor (ER) signaling, while DGCA/MEGENA and DICER tied in the number of modules significantly enriched in TNBC genes, and DICER identified the most modules significantly enriched in the KEGG terms Cell Cycle and Mismatch Repair. We then identified the modules unique to each method. We found the proportion of modules for each method that were not significantly enriched in any of the modules by one (off-diagonals, by column) or either (diagonals) of the other methods (Fig. 16b). DGCA/MEGENA identified a higher proportion of unique modules compared both pairwise and globally to the existing methods DiffCoEx and DICER.

## Discussion

The R package DGCA developed by this study is a powerful new tool for querying the regulatory relationship of gene pairs under different conditions. DGCA is applicable to a wide range of input data types, including microarray data, tissue-level or single-cell RNA-seq data, proteomic data, methylation data, and metabolomic data. In general, DGCA can be used to compare the correlations between features from both continuous- and count-based data types. Users input a gene expression matrix with gene identifiers in rows and samples in columns, a design matrix specifying which samples correspond to which conditions, and a vector specifying the conditions to be compared. In our differentially correlated gene pair simulation study, DGCA significantly outperformed EBcoexpress and Discordant in terms of accuracy and speed. When we applied DGCA to the RNA-seq data from the TCGA breast cancer samples with and without p53 mutations, we identified p53 pathway genes that had significant changes in correlation with *TP53* between the p53-mutated samples and those without p53 mutations. We revealed that p53 mutations altered the correlation patterns between the p53-pathway genes. We also studied the effect of non-silent PTEN DNA coding mutations, and showed that these mutations led to differential correlation between *PTEN* and two genes that it has been found to interact with. In the future, we will examine additional genes such as *MYC*, *KRAS*, and *ERBB2* in breast cancer and other cancers.

One of the limitations of our differential correlation approach to studying p53 mutations in breast cancer is that there is a wide variety in the functions of p53-mutations [53] and our approach averaged over many of them. As the sample size of the available data increases, it would be valuable to perform differential correlation on groups defined by individual p53 mutations or classes of p53 mutations. Given a larger sample size allowing for factorial analyses in differential correlation, it would also be interesting to consider both the effect of heterozygous deletions of the short arm of chromosome 17 that contain the p53 gene, which is often seen in combination with p53 coding mutations [77]. This would allow investigators to study whether *TP53* gene dosage affects p53 activity, especially because some but not all p53 coding mutations are able to act in a dominant-negative manner [78]. Another factor we will consider in future studies is *MDM2* gene amplification, which can also modulate the activity of p53 [54].

DGCA fits in well with the growing suite of tools available in the R statistical programming ecosystem for analyzing gene expression data, and can be used synergistically with a number of them. For example, as demonstrated empirically in this study, differential correlation is complementary to differential expression for discovering differences in gene expression between conditions. Differential correlation as a complement to differential expression is particularly apt in the case that the expression of a regulatory gene (e.g., a transcription factor) has its activity altered in one of the conditions without fully abrogating its expression, as is often the case for DNA mutations in tumor cells. However, differential correlation can be applied more broadly as well; for example, in discovering differences in the pathways that genes participate in between tissues and cell types. Differential correlation is also complementary to module detection approaches, such as MEGENA, as was shown in this manuscript through a comparison of estrogen receptor-positive breast cancer to triple-negative breast cancer. By using the finely-grained DGCA method, our approach was able to identify individual differential correlations between key gene pairs, and use them to create “bottom-up” differential correlation network modules. Further, differential correlation of individual gene pairs works particularly well downstream of higher-level module detection approaches, such as WGCNA, to perform “top-down” identification of modules with significant differential correlation. This is because parsing up the input gene expression matrix into smaller sets is often critical in order to make sense of the millions or even billions of gene pair combinations that can be analyzed in a typical RNA expression data set. In order to add further to the R programming gene expression analysis suite, future directions for improving DGCA include detection of linear changes in correlation across more than two conditions and integration with differential expression to define genes with differential wiring across conditions [79].

Aside from integration of DGCA and MEGENA, there are several alternative approaches available to identify modules of differentially correlated genes between conditions. One of the earliest studies to address this problem identified gene sets that led to the largest difference in an additive model that scored co-expression of genes in each condition [80]. This model-based approach allowed for an efficient search for gene sets that are co-expressed in one condition but not the other. A distinct approach called CoXpress first uses hierarchical clustering to identify groups of genes that are coexpressed across conditions, and then leverages sample permutations to measure whether each of these gene sets is significantly differentially coexpressed between conditions [12]. A separate approach called SDC (Subspace Differential Correlation) uses a biclustering approach to identify gene sets that are differentially coexpressed in subsets of each of two conditions [13]. DiffCoEx calculates dissimilarity scores between gene pairs in two or more conditions, and then uses leverages the WGCNA approach to identify modules based on this dissimilarity matrix [44]. An approach called DICER (Differential Correlation in Expression for meta-module Recovery) first calculates a probabilistic score for genewise differential correlation between conditions [10], and then performs hierarchical clustering on these differential correlation scores to identify modules of differential correlation, as well as meta-modules of modules that demonstrate differences in correlation across conditions. Another class of approaches also quantifies differential coexpression between conditions given an initial collection of gene sets, including Gene Set Co-expression Analysis (GSCA) [15] and Gene Sets Net Correlations Analysis (GSNCA) [16].

The module detection method described in this manuscript differs from all of these methods in that it does not set out solely to identify modules, but rather to identify individual gene pairs links with significant differentially correlations between conditions. Using this empirically identified set gene pairs, we demonstrated how integration with MEGENA, which has been previously shown to outperform alternatives in co-expression network construction [6], can be used to construct a planar filtered network and identify informative modules. This integration allows for visualization of the individual differential correlation links and their differential correlation classes, as well as the identification of hub genes within each module, which to the best of our knowledge none of the existing methods offer. Notably, DGCA is also complementary with several of the previously described approaches, since DGCA offers functions to calculate the average correlation difference across conditions within a module of genes, both averaging across all genes in the module and averaging across one gene compared to all other genes in the module.

We further sought to comprehensively assess the performance of the differential correlation modules identified by DGCA/MEGENA in comparison with the two most similar methods, DiffCoEx and DICER. First, in our simulation study for detecting differentially correlated modules, DGCA/MEGENA consistently outperformed DiffCoEx and DICER under the more general simulation settings, whereas DGCA/MEGENA has comparable performance with DiffCoEx and DICER under the more extreme circumstance in which a vast majority of gene pairs are differentially correlated. This result makes sense in light of the fact that DGCA/MEGENA adopts a “bottom-up” approach by first identifying differentially correlated gene pairs and then detecting modular structures, whereas DiffCoEx and DICER employ a “top-down” approach that relies more heavily on a consistent relationship between many gene pairs within a module across conditions. Therefore, DGCA/MEGENA works well under a wider range of circumstances where DiffCoEx and DICER miss many less densely connected modules. Next, we found that all three of the methods identified differential correlation modules that were significantly enriched for gene signatures related ER+ and triple-negative breast cancer subtypes, though DGCA/MEGENA and DICER identified the most relevant modules. A limitation of this analysis is that although enrichment of disease-relevant gene sets as a proxy for efficacy in different coexpression module detection has been used before [10], it is unclear whether a more sensitive approach to disease-associated module detection is necessarily better when considering the possibility of false positives. Therefore, the problem of optimal differential coexpression module detection on realistic biological data awaits future study, including the identification of a gold-standard data set to compare across methods in an unbiased and biologically meaningful way. However, DGCA/MEGENA identified the highest proportion of unique modules and this strongly suggests that DGCA/MEGENA does represent a novel method for differential correlation module detection. Thus, DGCA/MEGENA increases the diversity of options for this type of analysis.

As an important aspect of DGCA is the use of permutation samples to assess the statistical significance of differential correlations, we offer some guidelines for the use of this approach. It is important to distinguish between permutation analyses that use pooled empirical null distributions as opposed to analyses that use empirical null distributions from each gene or gene pair [81]. For pooled approaches, fewer permutations are needed because information is shared across gene pairs; for example, given a 10,000 gene data set and five permutations, the differential correlation of each actual gene pair is compared to 249,975,000 permuted gene pairs in order to estimate empirical null *p*-values. However, this vastly increases the memory footprint required for empirical *p*-value calculation and q-value estimation. Perhaps as a result, some investigators have used only one permutation of the data in such analyses [9]. If these memory constraints limit the number of permutations possible, we suggest that a reasonable approach is to repeat the analysis. For example, we repeated the ER+ vs TN breast cancer differential correlation analysis three times and found that the number of significantly identified differentially correlated gene pairs at both q < 0.05 and q < 0.01 were within acceptable limits (within 30% of one another) for the purpose of downstream analyses on gene sets. Although the above reasoning applies to cases with many gene pairs, heavy filtering of the input data and/or differential correlation calculations of only one gene compared to all others leads to far fewer empirical null statistics. For example, five permutations of the 295 gene pairs for the p53 differential correlation analysis would lead to only 1475 permuted gene pairs for use in estimating empirical null *p*-values, which is why we used more permutations (10,000) in this case. Non-pooled approaches are commonly used on the data sets with reduced dimensionality, such as the module level, or in our case, in the comparison of the average correlation of each gene compared to all other genes in two conditions. Non-pooled permutation approaches commonly use on the order of 100 or 1000 permutations [82, 83], and we use 1000 permutations to balance interpretability with computational efficiency. Notably, the number of permutations in non-pooled approaches delineates a clear lower bound on the empirical *p*-value that can be ascertained from the analysis. Overall, the number of permutations used in DGCA for pooled reference distributions depends on the total number of empirical gene pairs under consideration, while for non-pooled reference distributions it depends on the desired sensitivity of the lower bound of empirical *p*-values.

## Conclusions

Despite the theoretical advantages of studying differences in the correlation of key gene pairs between conditions, differential coexpression or differential correlation is not yet widely utilized, partially due to the lack of effective and biology-oriented software packages that enable biological meaningful findings. Our R package, DGCA, provides a comprehensive and user-friendly tool for not only calculating differential correlation between two conditions but also performing a number of downstream functional analyses including categorization of differential correlations, identification of multiscale differential correlation clustering structures, detection of key differential correlation hubs, and enrichment tests of functional pathways in differential correlation categories and clusters. Our differentially correlated gene pair and module detection simulation studies show that DGCA and DGCA/MEGENA perform favorably compared to the existing alternative methods. Our application to breast cancer data demonstrates that DGCA is capable of unlocking novel insights into real biological problems. This user-friendly, effective, and comprehensive software tool will greatly facilitate the application of differential correlation analysis in many biological studies and thus will help identification of novel signaling pathways, biomarkers, and targets in complex biological systems and diseases.

### Software and data availability

The DGCA R package will be available for download from CRAN (the Comprehensive R Archive Network, https://cran.r-project.org/), a repository of open-source software. Source code and other files are available at https://github.com/andymckenzie/DGCA.

Below is R code for using the DGCA package to analyze a subset of a publicly available single-cell RNA-seq data set.

- Step 1. Load the DGCA R package. > *library(DGCA)*
- Step 2. Read in the gene expression matrix. > *data(darmanis)*
- Step 3. Read in the design matrix. > data(design_mat)
- Step 4. Perform the basic differential correlation analysis for the gene RTN4 between oligodendrocytes and neurons. > *dgca_res = ddcorAll(inputMat = darmanis, design = design_mat, compare = c(“oligodendrocyte”, “neuron”), adjust = “perm”, nPerm = 10, splitSet = “RTN4”)*
- Step 5. View the top 20 genes differentially correlated with RTN4 as well as their differential correlation statistics. > *head(dgca_res, 20)*
- Step 6. Visualize the correlation in each condition between RTN4 and its top differentially correlated gene pair in this data set. > *plotCors(inputMat = darmanis, design = design_mat, compare = c(“oligodendrocyte”, “neuron”), geneA = “RTN4”, geneB = “COX6A1”)*
  For a more detailed explanation about package usage, please read the R help documents or vignettes. > *help(package = “DGCA”)*; *vignette(package = “DGCA”)*
  The Cancer Genome Atlas (TCGA) datasets supporting the conclusions of this article are available in the National Cancer Institute's Genomic Data Commons, https://gdc.cancer.gov/.

## Additional files

Additional file 1: Gene sets used in the enrichment analysis for the breast cancer modules. The five cleaned gene sets used for enrichment analysis of the differential correlation modules. (TSV 9 kb)
Additional file 2: Full table for differential correlation output from the p53 mutation study. The output of running DGCA on the p53 pathway gene set in the breast cancer RNA-seq samples, comparing correlations of these genes with *TP53* in non-mutated samples to correlations in p53-mutated samples, using the option to consider only positive correlations in calculate differential correlation between conditions. The “Classes” column indicates the correlation class of each of the genes where, e.g., “+/0” indicates a significant positive correlation in the non-mutated samples and no significant correlation in the p53-mutated samples. Note that the significance for the correlations within each condition is not adjusted for multiple comparisons. 10,000 permutation samples were generated in order to estimate empirical *p*-values, using a pooled reference distribution approach, from which q-values were calculated. WT = Wildtype, Mut. = Non-silent p53 mutation. (TSV 46 kb)
Additional file 3: Differential expression output from the p53 mutation study. The results of differential expression (using the R package limma) on the input breast cancer RNA-seq data filtered for genes in the p53-pathway. Note that the contrast called in limma used p53 non-mutated samples as the reference condition, so positive logFC values are upregulated in the mutant condition compared to wildtype, while negative logFC values are downregulated in the mutant condition compared to wildtype. (TSV 33 kb)
Additional file 4: Full table for average differential correlation output from the p53 mutation study. Genes are ranked by the significance of their median difference in correlation for each gene and all other genes in the p53 pathway gene set between non p53-mutated and p53-mutated breast cancer RNA-seq samples. 1000 permutation samples were generated in order to estimate empirical *p*-values for each gene, which were adjusted by the Benjamini-Hochberg method to control the false discovery rate among this set of genes. (TSV 13 kb)
Additional file 5: Full table for differential correlation output from the PTEN mutation study. The output of running DGCA on the PTEN pathway gene set in the breast cancer RNA-seq samples, comparing correlations of these genes with *PTEN* in non-mutated samples to correlations in PTEN-mutated samples. The “Classes” column indicates the correlation class of each of the genes where, e.g., “+/0” indicates a significant positive correlation (*p* < 0.05) in the non-mutated samples and no significant correlation in the PTEN -mutated samples. Note that the significance for the correlations within each condition is not adjusted for multiple comparisons. 10,000 permutation samples were generated in order to estimate empirical *p*-values, using a pooled reference distribution approach, from which q-values were calculated. WT = Wildtype, Mut. = Non-silent PTEN mutation. (TSV 10 kb)
Additional file 6: Differential expression output from the PTEN mutation study. The results of differential expression (using the R package limma) on the input breast cancer RNA-seq data filtered for genes in the PTEN-pathway. Note that the contrast called in limma used PTEN wildtype samples as the reference condition, so positive logFC values are upregulated in the mutant condition compared to wildtype, while negative logFC values are downregulated in the mutant condition compared to wildtype. (TSV 7 kb)
Additional file 7: Differential correlation modules from each method analyzed. Genes identified in each of the differential correlation modules with >25 and <1000 members by DGCA/MEGENA, DICER, and DiffCoEx from the same filtered ER+ vs TNBC RNA expression data set. (TSV 360 kb)

## Abbreviations

DGCA: Differential Gene Correlation Analysis (R package name)
dz: Difference in z-scores
ER+: Estrogen Receptor-Positive (breast cancer subtype)
FDR: False discovery rate
GOC: Gain of correlation
LOC: Loss of correlation
p53: tumor protein 53 protein isoform
TCGA: The Cancer Genome Atlas
TN: Triple Negative (breast cancer subtype)
TP53: Tumor Protein P53 gene.
